# Acupuncture Analgesia in Patients with Postoperative Neck Pain: A Protocol for Systematic Review and Meta-Analysis

**DOI:** 10.1155/2022/1226702

**Published:** 2022-07-20

**Authors:** Renming Liu, Songming Li, Yuhuan Liu, Min He, Jiazhen Cao, Mengmeng Sun, Changwei Duan, Tie Li

**Affiliations:** ^1^Department of Acupuncture and Moxibustion, Changchun University of Chinese Medicine, Changchun, Jilin 130117, China; ^2^The 3rd Affiliated Hospital of Changchun University of Chinese Medicine, Changchun, Jilin 130118, China; ^3^Northeast Asian Institute of Traditional Chinese Medicine, Changchun University of Chinese Medicine, Changchun, Jilin 130021, China

## Abstract

*Background*. There is a yearly increase in pain after neck surgery, which is accompanied by high consumption of opioids. However, the opioid addiction epidemic is one of the most serious public health problems worldwide. Therefore, it is important to find suitable alternatives for opioids. Acupuncture therapy has been found effective for some types of pain control. This protocol aims to evaluate the efficacy and safety of acupuncture therapy in the treatment of pain after neck surgery. *Methods and Analysis.* We will search eight electronic databases from their inception to April 2022. Only randomized controlled trials (RCTs) using manual acupuncture, auricular acupuncture, or electroacupuncture as major therapy will be included, regardless of whether the study was published in Chinese or English. The selection of studies and data extraction will be independently completed by at least two experienced reviewers with a master's degree. The methodological quality of the included studies will be assessed by the Cochrane risk-of-bias tool. For the meta-analysis, Review Manager Statistical (RevMan V.5.3) software will be used. The results will be presented as the risk ratio (RR) for the binary data and the mean difference (MD) or standardized mean difference (SMD) for the continuous data. *Ethics and Dissemination.* This protocol for a systematic review will be submitted to a peer-reviewed journal for publication and presented at a relevant conference, and there is no need to obtain formal ethical approval. *Trial Registration Number.* PROSPERO registration number CRD42021281722.

## 1. Introduction

Adequate postoperative pain management is important for successful recovery and rehabilitation after surgery [1]. Neck surgery mainly involves tonsillectomy, thyroidectomy, or parathyroidectomy, and neck dissection. Depending on the type of surgical intervention, 25%–65% of patients have moderate to severe postdischarge pain, leading to their dissatisfaction with postoperative care [2]. Tonsillectomy is one of the most common surgical procedures. Each year, 500,000 individuals in the United States undergo tonsillectomy [3], which is often performed during the daytime in an ambulatory setting by otolaryngologists and pediatricians [4]. Although the *French Oto-Rhino-Laryngology Head and Neck Surgery Society* [5] and other researchers [6] have published guidelines for post-tonsillectomy pain management in adults and children, respectively, pain management remains challenging and poorly managed in clinical practice [7]. Postoperative pain after tonsillectomy is related to the indications and surgical techniques [8]. In adults, pain is always undertreated, and the type of surgery requires dissection with coagulation, which leads to severe pain [9]. In children, the most common issue is low parent and child adherence, and about 60% of children receive less medication during postoperative days than prescribed [10]. Therefore, complementary and alternative medicine (CAM) is needed to manage their pain.

In the United States, approximately 20,000 thyroidectomies and parathyroidectomies are performed each year [11, 12]. Every year, the total burden of new head and neck cancers exceeds 10,000 new cases [13]. Opioid analgesics are the most common prescription medications used to treat postoperative pain by the surgical team. However, they may result in opioid addiction [14]. Considering the continuous increase in the number of patients, better postoperative pain management is required.

Neck dissection includes the removal of lymph nodes from the neck [15] and remains the key component of the management of neck tumors [16]. In addition to lymph node removal, it also often involves the excision of the accessory nerve (CN XI), sternocleidomastoid muscle, and internal jugular vein. Therefore, neck dissection not only leads to neck dysfunction but also to neck pain [17]. Around 30% to 70% of patients may even experience variable degrees of shoulder pain [18–20]. Hence, their quality of life is substantially affected. Although physical therapy exercises and anti-inflammatory drugs are widely used to relieve the pain after neck dissection, their efficacy is often disappointing.

An increasing number of recent studies have focused on the use of acupuncture to treat postoperative pain after tonsillectomy, thyroidectomy or parathyroidectomy, and neck dissection. The positive effects of acupuncture therapy have been mentioned in acute postoperative pain management [21]; however, there is still relatively limited progress in therapeutics for effective symptom control. Although several systematic reviews of acupuncture for postoperative pain were published from 2015 to 2020 [22–25], there is still a lack of systematic reviews and meta-analyses of acupuncture for the treatment of pain after neck surgery. Therefore, a protocol for comprehensive research on pain management is of high priority. Moreover, few studies have revealed differences in efficacy between the acupuncture therapy and the conventional treatment of postoperative neck pain.

### 1.1. Description of the Intervention

Acupuncture therapy is an important part of physiotherapy and has a long history in China. It is based on the concept of vital energy. The United States Food and Drug Administration (FDA) approved acupuncture needles as a medical device in 1996 [26]. Acupuncture has been suggested for some postoperative symptoms by the National Institutes of Health (NIH) [27]. As time goes by, the types of acupuncture therapy have gradually increased, and a large number of acupuncture methods are accepted in clinics, such as manual acupuncture (MA), auricular acupuncture (AA), and electroacupuncture (EA). Acupuncture therapy is effective for a variety of painful conditions [28]. Furthermore, it can be used in the perioperative period, and it exerts its effects at three different levels including the peripheral site, the spinal cord, and supraspinal structures [29]. Therefore, a more specific type of pain needs further study, and the most effective acupuncture method needs to be determined.

## 2. Materials and Methods

We used the PRISMA-P checklist when writing our report (PRISMA-P) [30]. The review will be conducted as per the PRISMA statement guidelines [31]. The protocol we have registered at PROSPERO is available on the website at https://www.crd.york.ac.uk/prospero/.

### 2.1. Types of Studies

We will search for relevant randomized controlled trials (RCTs) published from the inception date of the databases to April 2022, without any regional limitations. Both articles published in English and Chinese will be considered. Only RCTs will be included, whereas animal studies, meeting abstracts, case reports, case series, editorials, protocols, and comments will be excluded.

### 2.2. Types of Participants

The population of interest consists of adult patients (aged more than 18 years old) who underwent postoperative neck pain after tonsillectomy, thyroidectomy, or parathyroidectomy, or neck dissection. All eligible participants will be included regardless of age, race, gender, ethnic background, nationality, economic status, and source of cases.

### 2.3. Types of Intervention

We will restrict our focus to studies that used different methods of acupuncture treatment as a primary intervention. The following types of acupuncture methods will be eligible: (1) manual acupuncture (MA), which is a part of traditional Chinese medicine; in MA, pain is alleviated by inserting the needles into specific points of the body, and the mechanism seems to involve the central nervous system; (2) auricular acupuncture (AA), which mainly stimulates the acupoints of the ear and relieves pain with the pressure of the Vaccaria seeds; and (3) electroacupuncture (EA), which has been further developed based on the traditional acupuncture theory; its function is a transformation of energy. Any combination of these acupuncture types will also be included. The duration of the study research will not be restricted in our meta-analysis. However, other irrelevant needle stimulation of acupoints, such as cupping, laser acupuncture, or acupotomy, will not be considered.

### 2.4. Types of Comparator(s)/Control

We will evaluate the following comparisons:

1. Acupuncture versus standard care (standard postoperative analgesic treatment)
2. Auricular acupuncture with stickers versus stickers alone, or versus without receiving adhesive tapes or stickers
3. Acupuncture versus placebo or sham acupuncture

We will exclude trials including combination therapy.

### 2.5. Types of Outcome Measures

#### 2.5.1. Primary Outcomes

The main goal of this study is to evaluate the efficacy of different acupuncture methods and find the best treatment time. Therefore, levels of pain intensity will be an important factor, and the primary outcome indicators will include the visual analog scale (VAS) [32] or the Constant–Murley score (CMS) [33], a composite score of pain.

#### 2.5.2. Secondary Outcomes

Secondary outcomes will include the following:

1. The Neck Dissection Impairment Index (NDII)
2. Numerical Rating Scale of Pain (NRS)
3. Modified Constant–Murley score
4. McGill Pain Questionnaire on postoperative days
5. Incidence of vomiting, nausea, and agitation

### 2.6. Search Strategy

#### 2.6.1. Electronic Search

Four English-language databases (PubMed, Embase, Web of Science, and Cochrane) and four Chinese-language databases (China National Knowledge Infrastructure, CBM, VIP Database for Chinese Technical Periodicals, and WANFANG) will be searched for RCTs published from the database's inception up to April 2022.

The search strategy will consist of three components: clinical condition (neck-dissection, tonsillectomy, thyroid, and parathyroid surgery); postoperative condition (postoperative pain, hyperalgesia, and allodynia); and intervention (manual acupuncture, electroacupuncture, and auricular acupuncture). We will use a combination of related terms and subject headings to retrieve relevant studies. The search strategy for the PubMed database is shown in Table 1.

#### 2.6.2. Searching Other Resources

We will also search electronically the World Health Organization (WHO) International Clinical Trial Registry Platform, the National Institute of Health (NIH) clinical registry Clinical Trials, the Chinese clinical registry, and the Australian New Zealand Clinical Trials Registry. The selected studies will be screened. Moreover, grey literature (not formally published by commercial or academic publishers) will be manually searched [34]. For ongoing or unpublished RCTs, we will contact the author of the trial to obtain the latest clinical data. In addition, we will consult the experts for some potential studies and unavailable clinical data.

### 2.7. Data Collection and Analysis

#### 2.7.1. Selection of Studies

**Figure 1:**
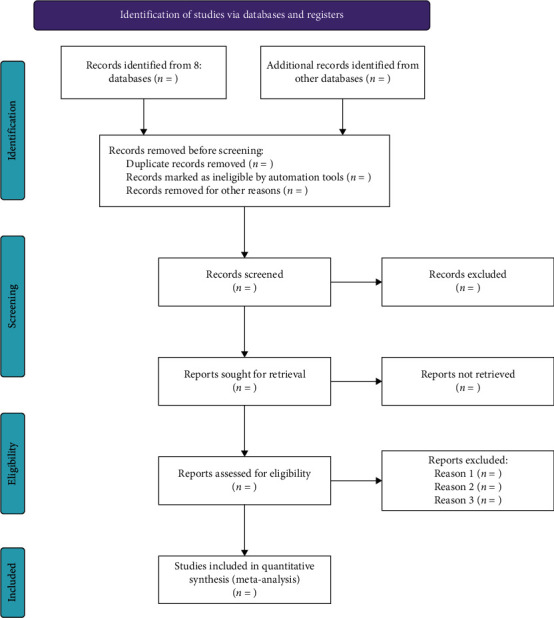
PRISMA 2020 flow diagram for a new systematic review and meta-analysis, which includes a search of databases and registers only.

First, one reviewer will use the software (EndNote X9) to import the search results and filter out the repetitive articles according to the designated strategies. Then, all of the extracted articles will be screened independently by two reviewers (JC and CD), who will examine the title, abstract, and keywords after professional training. If there are any disagreements, a third person (TL) will arbitrate. The potentially eligible full-text articles will be downloaded and screened by two reviewers (JC and CD). Later, EndNote X9 will be used again for the management of articles. When an article is excluded, the detailed reason will be recorded. The literature selection procedure is shown in Figure 1.

#### 2.7.2. Data Extraction and Management

When the search procedure is completed, two authors (RL and SL) will independently make pilot-tested data forms to complete the screening procedure. First, duplicate literature will be excluded. Second, according to the content of the title, abstract, and full text, the compliant studies will be retained. The following information will be recorded: (1) general information (title, the first author's name, nationality, year of publication, and journal name); (2) study design (random sequence generation, allocation concealment method, blinding method, conflict of interest, sex ratio of treatment group, sex ratio of control group, age of treatment group, age of control group, and sample size); (3) intervention and comparator (type of acupuncture therapy, acupoints selection, stimulation duration, needle depths and frequency, treatment duration, follow-up duration, and details of the control group); (4) outcomes (different types of outcomes and related statistical results); (5) patients' adverse reactions; and (6) funding. In the case of insufficient available data, information will be obtained by contacting the authors or by calculations based on our previous research. If any discrepancy happens, a third reviewer (CD) will make an adjudication.

### 2.8. Assessment of Risk of Bias

To assess the risk of bias, we will use the Cochrane Collaboration risk-of-bias tool appraised by two reviewers [35]. The risk of bias in sequence generation involves random sequence generation, allocation concealment, blinding of participants, blinding of outcome assessment, selective outcome reporting, and incomplete outcome data. Each domain will be categorized into the following three levels: low risk, unclear risk, and high risk. Any disagreement between the two reviewers will be resolved by the third reviewer (TL) through discussion.

### 2.9. Measures of Treatment Effect

The RevMan software V.5.3 will be used for efficacy data, including data synthesized and statistically analyzed. As an efficacy index of continuous variables, we will use the mean difference (MD) and SMD with 95% CIs. For categorical variables, we will use the risk ratio (RR) with 95% CIs for calculation.

### 2.10. Dealing with Missing Data

To acquire insufficient details and missing data for the selected articles, the corresponding authors or relevant authors of the articles will be contacted. If we do not receive a response and the required data are still unobtainable, imputation will not be performed for the missing data. To avoid additional bias introduction, we will only analyze the available data.

### 2.11. Assessment of Heterogeneity

The assessment of clinical and methodological heterogeneity will mainly focus on the characteristics of patients, interventions, and kinds of outcomes, and it will make a comparison of the goodness of fit between the fixed-effects model and the random-effects model. The *I*^2^ statistic, which derives from the *X*^2^ test, will be used to assess heterogeneity across the studies according to the Cochrane Handbook for Systematic Reviews of Interventions. If *I*^2^ < 50% and *p* > 0.1, the heterogeneity tests will show little or no statistical heterogeneity, and a fixed-effects model will be considered. In contrast, if *I*^2^ > 50% and *p* < 0.1, the heterogeneity tests will indicate high heterogeneity, and the random-effects model will be adopted. Specifically, much more attention should be paid to the source of heterogeneity if *I*^2^ ≥ 75%. Moreover, to explore the possible causes of heterogeneity, a subgroup analysis or meta-regression will be performed based on clinical characteristics.

### 2.12. Reporting Bias Assessment

To make sure that the results of the study are credible, reporting bias assessment will be necessary. If more than 10 trials are included [36], Begg's and Egger's tests will be used to assess the symmetry of the funnel plot by the Stata V.14.0 software.

### 2.13. Data Synthesis

We will use the Review Manager V.5.3 to conduct data processing from the Cochrane Collaboration. If there is little or no heterogeneity among the trials, the fixed-effects model will be used. In contrast, the random-effects model will be used for data synthesis if significant heterogeneity is shown (*I*^2^ ≥ 50%). Subgroup analysis will be carefully considered if necessary. Although a descriptive analysis will be provided, we will not conduct a meta-analysis if the heterogeneity is too large [37].

### 2.14. Subgroup and Sensitivity Analysis

In this study, subgroup analyses will be performed based on the treatment duration, follow-up time points, surgery types, acupoint selection, or different acupuncture methods. However, the ultimate grouping will be determined depending on the inclusion of studies providing relevant data. To make sure that the conclusions are credible, we will conduct a sensitivity analysis to verify the stability and reliability of the primary outcome in terms of the following three aspects: sample size, missing data, and statistical model.

### 2.15. Summary of Evidence

We will use the Grading of Recommendations Assessment, Development, and Evaluation (GRADE) approach to summarize the meta-analysis and grade the strength of evidence [35]. The GRADE Profiler evaluates the quality of evidence (risk of bias, heterogeneity, inconsistency, indirectness, imprecision, and publication bias), and the level will be rated as “high,” “moderate,” “low,” or “very low” [38].

## 3. Discussion

With the development of society, people are generally under a state of high pressure, which leads to some serious mood-related diseases such as tonsillitis, thyroid cancer, and neck neoplasms. For treating these diseases, there are few options besides surgery. Furthermore, most doctors and nurses pay much attention to the operation period rather than to postoperative pain management. The postoperative neck pain may not only cause patients' suffering but may also lead to an addiction to opioids. Many studies have shown that acupuncture therapy is effective for postoperative neck pain [39–42] and that it may replace opioids for postoperative analgesia. However, the treatment duration, the choice of acupoints, and even the pain rating scale are quite different. Furthermore, there are currently no systematic reviews on this topic. This meta-analysis will make a comparison between different types of acupuncture and objectively infer their efficacy. Thus, this study may offer a basis for replacing opioids for postoperative neck pain management and provide a novel regime for acupuncture practice.

Although there have been some meta-studies on acupuncture for pain after tonsillectomy [43–46], a relatively strict and scientific protocol design for such a study on the comprehensive assessment of acupuncture for postoperative neck pain has not been reported yet and needs to be prioritized. The results of this study will offer doctors and patients more available options. Data collection and management will be independently conducted by more than two trained researchers to ensure the objectivity of the study. However, there will be some limitations, considering that our proposed methodology will mainly focus on the stimulation of acupoints by needles, while we will place less emphasis on the associated selection of acupoints and different ways to stimulate the acupoints. In summary, this protocol will be updated in the future if needed, and the details of the changes will be added as a supplement.

## Figures and Tables

**Table 1:** Search strategy used in PubMed database.

Number	Search terms
1	MeSH descriptor: [neck dissection] explode all trees
2	(Tonsillectom^∗^):ab,ti, kw or (thyroidectom^∗^):ab,ti, kw or (parathyroidectom^∗^):ab,ti,kw
3	1 or 2
4	MeSH descriptor: [pain, postoperative] explode all trees
5	(Postsurgical pain):ab,ti, kw or (postoperative pain)ab,ti, kw or (perioperative period):ab,ti,kw
6	4 or 5
7	MeSH descriptor: [hyperalgesia] explode all trees
8	(Hyperalg^∗^):ab,ti, kw or (hyperalgesic sensation^∗^):ab,ti, kw or (secondary hyperalg^∗^):ab,ti, kw or (hyperalgesia^∗^, thermal):ab,ti, kw or (primary hyperalgia^∗^):ab,ti, kw or (mechanical hyperalg^∗^):ab,ti, kw or (allodynia, thermal):ab,ti, kw or (allodynia^∗^):ab,ti, kw or (tactile allodynia):ab,ti, kw or (mechanical allodynia):ab,ti,kw
9	7 or 8
10	6 or 9
11	MeSH descriptor: [acupuncture therapy] explode all trees
12	(Acupuncture treatment^∗^):ab.ti.kw or (pharmacoacupuncture treatment):ab,ti, kw or (pharmacoacupuncture Therapy):ab,ti, kw or (acupotom^∗^):ab,ti,kw
13	11 or 12
14	MeSH descriptor: [acupuncture, ear] explode all trees
15	(Ear acupuncture^∗^):ab,ti, kw or (auricular acupuncture^∗^):ab,ti,kw
16	14 or 15
17	MeSH descriptor: [electroacupuncture] explode all trees
18	13 or 16 or 17
19	3 and 10 and 18

## Data Availability

No data were used to support this study.

## References

[1] Lovich-Sapola J., Smith C. E., Brandt C. P. (2015). Postoperative pain control. *Surgical Clinics of North America*.

[2] Shoqirat N., Mahasneh D., Al-Khawaldeh O., Singh C. (2019). Postoperative patients in jordan: pain prevalence, characteristics, beliefs, and satisfaction. *Pain Management Nursing*.

[3] Chaturvedi A. K., Song H., Rosenberg P. S. (2016). Tonsillectomy and incidence of oropharyngeal cancers. *Cancer Epidemiology, Biomarkers & Prevention*.

[4] Crowson M. G., Ryan M. A., Rocke D. J., Raynor E. M., Puscas L. (Mar. 2017). Variation in tonsillectomy rates by health care system type. *International Journal of Pediatric Otorhinolaryngology*.

[5] Gerbershagen P. D. H. J., Aduckathil S., van Wijck A. J. M., Peelen L. M., Kalkman C. J., Meissner W. (2013). Pain. *Pain Medications*.

[6] Aldamluji N., Burgess A., Pogatzki-Zahn E., Raeder J., Beloeil H., PROSPECT Working Group collaborators (2021). PROSPECT guideline for tonsillectomy: systematic review and procedure-specific postoperative pain management recommendations. *Anaesthesia*.

[7] Persino P. R., Saleh L., Walner D. L. (2017). Pain control following tonsillectomy in children: a survey of patients. *International Journal of Pediatric Otorhinolaryngology*.

[8] Sarny S., Habermann W., Ossimitz G., Stammberger H. (2012). Significant post-tonsillectomy pain is associated with increased risk of hemorrhage. *Annals of Otology, Rhinology & Laryngology*.

[9] Lee Y.-C., Hsin L.-J., Lin W.-N., Fang T. J., Tsai Y. T., Luo C. M. (2020). Adolescents and adults undergoing temperature-controlled surgical instruments vs electrocautery in tonsillectomy. *JAMA Otolaryngology–Head & Neck Surgery*.

[10] Stewart D. W., Ragg P. G., Sheppard S., Chalkiadis G. A. (2012). The severity and duration of postoperative pain and analgesia requirements in children after tonsillectomy, orchidopexy, or inguinal hernia repair. *Pediatric Anesthesia*.

[11] Sun G. H., Demonner S., Davis M. M. (2013). Epidemiological and economic trends in inpatient and outpatient thyroidectomy in the United States, 1996–2006. *Thyroid*.

[12] Kim S. M., Shu A. D., Long J. (2016). Declining rates of inpatient parathyroidectomy for primary hyperparathyroidism in the US. *PLoS One*.

[13] Siegel R. L., Miller K. D., Jemal A. (2019). Cancer statistics, 2019. *CA: A Cancer Journal for Clinicians*.

[14] Cramer J. D., Wisler B., Gouveia C. J. (2018). Opioid stewardship in otolaryngology: state of the art review. *Otolaryngology—Head and Neck Surgery (United States)*.

[15] Orloff L. A., Kuppersmith R. B. (2010). American thyroid association’s central neck dissection terminology and classification for thyroid cancer consensus statement. *Otolaryngology-Head and Neck Surgery*.

[16] Deganello A., Battat N., Muratori E. (2016). Acupuncture in shoulder pain and functional impairment after neck dissection: a prospective randomized pilot study. *Laryngoscope*.

[17] Gane E. M., Michaleff Z. A., Cottrell M. A. (2017). Prevalence, incidence, and risk factors for shoulder and neck dysfunction after neck dissection: a systematic review. *European Journal of Surgical Oncology*.

[18] Gane E. M., O’Leary S. P., Hatton A. L., Panizza B. J., McPhail S. M. (2017). Neck and upper limb dysfunction in patients following neck dissection: looking beyond the shoulder. *Otolaryngology—Head and Neck Surgery*.

[19] Trivić A. S., Djukić V. B., Krejović-Trivić S. B., Milovanovic J., Stankovic P., Milovanovic A. (2009). Postoperative morbidity and quality of live in patients after radical and modified radical neck dissection. *Acta Chirurgica Iugoslavica*.

[20] Barber B., McNeely M., Chan K. M. (2015). Intraoperative brief electrical stimulation (BES) for prevention of shoulder dysfunction after oncologic neck dissection: study protocol for a randomized controlled trial. *Trials*.

[21] Mitra S., Carlyle D., Kodumudi G., Kodumudi V., Vadivelu N. (2018). New advances in acute postoperative pain management. *Current Pain and Headache Reports*.

[22] Wu M.-S., Chen K.-H., Chen I.-F. (2016). The efficacy of acupuncture in post-operative pain management: a systematic review and meta-analysis. *PLoS One*.

[23] Lee A., Fan L. T. Y. (2009). Stimulation of the wrist acupuncture point P6 for preventing postoperative nausea and vomiting. *Cochrane Database of Systematic Reviews*.

[24] Yuan W., Wang Q. (2019). Perioperative acupuncture medicine. *Chinese Medical Journal*.

[25] Zhao Y., Zhang L., Wang Y. (2020). Acupuncture therapy for postoperative pain of anorectal diseases. *Medicine (Baltimore)*.

[26] Eskinazi D. P., Jobst K. A. (1996). Editorial. *Journal of Alternative & Complementary Medicine*.

[27] (1998). NIH consensus conference. Acupuncture. *JAMA*.

[28] Kelly R. B., Willis J. (2019). Acupuncture for pain. *American Family Physician*.

[29] Zhang R., Lao L., Ren K., Berman B. M. (2014). Mechanisms of acupuncture-electroacupuncture on persistent pain. *Anesthesiology*.

[30] Moher D., Shamseer L., Clarke M. (2015). Preferred reporting items for systematic review and meta-analysis protocols (PRISMA-P) 2015 statement. *Systematic Reviews*.

[31] Hutton B., Salanti G., Caldwell D. M. (2015). The PRISMA extension statement for reporting of systematic reviews incorporating network meta-analyses of health care interventions: checklist and explanations. *Annals of Internal Medicine*.

[32] Chiarotto A., Maxwell L. J., Ostelo R. W., Boers M., Tugwell P., Terwee C. B. (2019). Measurement properties of visual analogue scale, numeric rating scale, and pain severity subscale of the brief pain inventory in patients with low back pain: a systematic review. *Journal of Pain*.

[33] Barreto R. P. G., Barbosa M. L. L., Balbinotti M. A. A., Mothes F. C., da Rosa L. H. T., Silva M. F. (2016). The Brazilian version of the constant–murley score (CMS-BR): convergent and construct validity, internal consistency, and unidimensionality. *Revista Brasileira de Ortopedia (English Edition)*.

[34] Monaghan C., Linden B., Stuart H. (2021). Postsecondary mental health policy in Canada: a scoping review of the grey literature: politique de santé mentale post-secondaire au Canada: un examen de la portée de la littérature grise. *Canadian Journal of Psychiatry*.

[35] Cumpston M., Li T., Page M. J. (2019). Updated guidance for trusted systematic reviews: a new edition of the cochrane handbook for systematic reviews of Interventions. *Cochrane Database of Systematic Reviews*.

[36] Ren R., Zhang J., Zhang T., Peng Y., Tang C., Zhang Q. (2019). Auriculotherapy for sleep quality in people with primary insomnia: a protocol for a systematic review and meta-analysis. *Medicine (Baltimore)*.

[37] Zhou J., Peng W., Li W., Liu Z. (2014). Acupuncture for patients with alzheimer’s disease: a systematic review protocol. *BMJ Open*.

[38] Guyatt G. H., Oxman A. D., Schünemann H. J., Tugwell P., Knottnerus A. (2011). GRADE guidelines: a new series of articles in the journal of clinical epidemiology. *Journal of Clinical Epidemiology*.

[39] Pfister D. G., Cassileth B. R., Deng G. E. (2010). Acupuncture for pain and dysfunction after neck dissection: results of a randomized controlled trial. *Journal of Clinical Oncology*.

[40] Shah A. N., Moore C. B., Brigger M. T. (2020). Auricular acupuncture for adult tonsillectomy. *Laryngoscope*.

[41] Dingemann J., Plewig B., Baumann I., Plinkert P. K., Sertel S. (2017). Einsatz von akupunktur beim post-tonsillektomieschmerz: eine prospektive, randomisierte und kontrollierte doppelblindstudie. *HNO*.

[42] Sertel S., Herrmann S., Greten H. J. (2009). Additional use of acupuncture to NSAID effectively reduces post-tonsillectomy pain. *European Archives of Oto-Rhino-Laryngology*.

[43] Kahn C. I., Huestis M. J., Cohen M. B., Levi J. R. (2020). Evaluation of acupuncture’s efficacy within otolaryngology. *Annals of Otology, Rhinology & Laryngology*.

[44] Pouy S., Etebarian A., Azizi-Qadikolaee A., Saeidi S. (2019). The effect of acupuncture on postoperative pain, nausea and vomiting after pediatric tonsillectomy: a systematic review. *International Journal of Adolescent Medicine and Health*.

[45] Shin H. C., Kim J. S., Lee S. K. (2016). The effect of acupuncture on postoperative nausea and vomiting after pediatric tonsillectomy: a meta-analysis and systematic review. *Laryngoscope*.

[46] Cho H. K., Park I. J., Jeong Y. M., Lee Y. J., Hwang S. H. (2016). Can perioperative acupuncture reduce the pain and vomiting experienced after tonsillectomy? a meta-analysis. *Laryngoscope*.

